# Effects of inhaling essential oils of *Citrus limonum L*., *Santalum album*, and *Cinnamomum camphora* on human brain activity

**DOI:** 10.1002/brb3.2889

**Published:** 2023-01-09

**Authors:** Kazutaka Ueda, Tatsushi Horita, Takeshi Suzuki

**Affiliations:** ^1^ Department of Mechanical Engineering, Graduate School of Engineering The University of Tokyo Tokyo Japan; ^2^ Laboratory Nippon Kodo Co., Ltd. Tokyo Japan

**Keywords:** *Cinnamomum camphora*, *Citrus limonum* *L*, essential oil, human brain, *Santalum album*

## Abstract

**Introduction:**

Essential oil inhalation has various effects on the human body. However, its effects on cognitive function and the neural basis remain unclear. We aimed to investigate the effects of inhaling lemon, sandalwood, and kusunoki essential oils on human brain activity and memory function using multichannel electroencephalography and brain source activity estimation.

**Methods:**

Participants performed a letter 2‐back working memory task during electroencephalography measurements before and after essential oil inhalation. Brain activation, task difficulty, concentration degree, and task performance were compared among the essential oils and a fragrance‐free control.

**Results:**

Task performance significantly improved after lemon essential oil inhalation. Lemon essential oil inhalation resulted in delta and theta band activation in the prefrontal cortex, including the anterior cingulate gyrus and orbitofrontal cortex, superior temporal gyrus, parahippocampal gyrus, and insula. During inhalation, persistent alpha band activation was observed in the prefrontal cortex, including the anterior cingulate gyrus. Sandalwood essential oil inhalation led to beta and gamma band activation in the prefrontal cortex, including the anterior cingulate gyrus.

**Conclusion:**

Our findings demonstrate that different essential oils have specific effects on brain activity related to emotion and memory processing.

## Graphical abstract

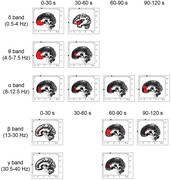

## 1 INTRODUCTION

Inhaling essential oils reduces stress (Chamine & Oken, 2016; Heuberger et al., 2006; Höferl et al., 2016; Kim et al., 2011; Motomura et al., 2001; Shimada et al., 2011; Toda & Morimoto, 2008), maintains concentration (Ho & Spence, 2005; Kaneki et al., 2005), and improves sleep (Fismer & Pilkington, 2012; Hirokawa et al., 2012) and dementia symptoms (Ballard et al., 2002; Holt et al., 2003; Smallwood et al., 2001) in humans. Human cognitive functions, including perception, attention, memory, language, thought, and emotion, collectively facilitate higher‐order processing, including decision making and creativity. The effects of essential oil inhalation on these functions and their neural basis remain unclear. A study on the relationship between essential oil inhalation and cognitive function (Moss et al., 2008) reported that inhaling peppermint oil improved memory function. Moreover, an electroencephalography (EEG) study reported that inhaling lavender oil significantly attenuated alpha band (8−10 Hz) EEG activity in the parietal and posterior temporal brain regions (Masago et al., 2000). Moreover, inhaling lavender oil significantly increased beta band (21−30 Hz) EEG activity in the frontal region (Diego et al., 1998). Although some studies have reported on the effects of lemon oil inhalation on stress (Komiya et al., 2006) and pain reduction (Ikeda et al., 2014) and on the neural mechanisms underlying these effects, they were mostly conducted on rodents. Previous studies have used physiological indices associated with the autonomic nervous system, including heart rate and skin conductance response, to analyze the stress‐reducing effects of sandalwood oil inhalation (Heuberger et al., 2006; Höferl et al., 2016); however, none reported on cognitive function and human brain activity.

The limbic system, which is close to the olfactory nerve, is considered the most important pathway for direct signal transmission from the olfactory nerve to the brain after intranasal absorption (Kandel et al., 2012). The limbic system comprises the hippocampus, which controls memory functions (Burgess et al., 2002; Phelps, 2004), and the amygdala (Davis & Whalen, 2001; Phelps, 2004) and anterior cingulate cortex (Bush et al., 2000), which control emotional functions. Therefore, essential oil inhalation may influence memory and emotional functions in humans. In this study, we focused on memory function while examining the effects of essential oil inhalation on cognitive function and human brain activity.

EEG measurements acquired at high temporal resolutions can reveal the time course of brain activation after essential oil inhalation. Moreover, multichannel head‐surface EEG with exact low‐resolution brain electromagnetic tomography (Pascual‐Marqui et al., 2011; Pascual‐Marqui et al., 1994) can facilitate brain source activity estimation, including that within deep brain regions, such as the hippocampus and anterior cingulate cortex (Cannon et al., 2005; Pizzagalli et al., 2004).

Here, we aimed to evaluate the effects of inhaling lemon, sandalwood, and kusunoki (i.e., camphor) essential oils on human brain activity and memory function using EEG and a working memory task. Numerous brain regions are involved in working memory. Additionally, specific EEG frequencies are involved in human working memory functions. Working memory activity in the delta band could be associated with the frontal lobe (de Vries et al., 2018; Zarjam et al., 2011) and parahippocampal gyrus (Imperatori et al., 2013). Moreover, numerous studies have demonstrated the importance of the theta band in the prefrontal cortex, especially in the medial prefrontal cortex (Gevins et al., 1998; Hsieh & Ranganath, 2014; Jensen & Tesche, 2002; Meltzer et al., 2008; Onton et al., 2005; Sauseng et al., 2010). Additionally, EEG activity in the alpha (Meltzer et al., 2008; Sauseng et al., 2010), beta (Tallon‐Baudry et al., 1999), and gamma (Meltzer et al., 2008; Roberts et al., 2013; Roux & Uhlhaas, 2014) bands in the prefrontal cortex increases in association with working memory. We hypothesized that essential oil inhalation would activate EEG signals in the frequency bands of the brain regions involved in working memory task performance. This is the first study to investigate the effects of essential oil inhalation on the human brain, including the deep regions, using a memory demanding task.

## 2 MATERIALS AND METHODS

### 2.1 Participants

We included 24 healthy men (mean age ± standard deviation: 21.88 ± 1.57 years) without self‐reported abnormalities in the olfactory senses. All participants were right‐handed according to the Flinders handedness survey (Okubo et al., 2014). For the experiment, participants were instructed not to wear anything containing fragrances (e.g., perfume, cosmetics). Moreover, they were instructed not to eat or drink anything with strong aromas (e.g., gum, curry, garlic, coffee, tea). This study was approved by the Ethics Committee of the Graduate School of Engineering, The University of Tokyo (approval number: KE20‐85), and was conducted in accordance with the Declaration of Helsinki. All participants provided written informed consent.

### 2.2 Stimuli

We used lemon, sandalwood, and kusunoki essential oils at a concentration of 10% in dipropylene glycol (DPG). The authors, including perfumers and fragrance researchers, determined the concentrations at which all the essential oils could be perceived at a similar intensity. Lemon essential oil was obtained by cold pressing Italian lemon peels (*Citrus limonum L*.). Sandalwood essential oil was obtained from a sandalwood log (*Santalum album*) through supercritical extraction in South India. Kusunoki essential oil was obtained from Japanese kusunoki wood (*Cinnamomum camphora*) by steam distillation. Moreover, we used DPG‐only samples without added fragrances as controls. All liquids were transparent and indistinguishable based on appearance. The participants inhaled the fragrances from cut cotton containing 50 μg of each essential oil. The authors preliminarily inhaled several quantities of the essential oils applied to cut cotton and determined that 50 μg of oil could be smelled adequately and was not too strong.

### 2.3 Apparatus

A 29.8″ display (MultiSync LCD‐PA302W, NEC Corp, Tokyo, Japan; effective display area of 641 × 401 mm^2^) and mouse were set up on a desk. The participants were seated on a chair in front of the desk and performed the task using the mouse with their right hand. During essential oil inhalation, the participants’ chin and forehead were fixed on a chinrest to which the cut cotton containing the oil was attached (distance between nose tip and cotton was approximately 1 cm). The software application, Presentation^®^ (Neurobehavioral Systems, Inc., Berkley, CA, USA), was used to control the experimental task presentation and participants’ mouse input.

### 2.4 Experimental task

**FIGURE 1:**
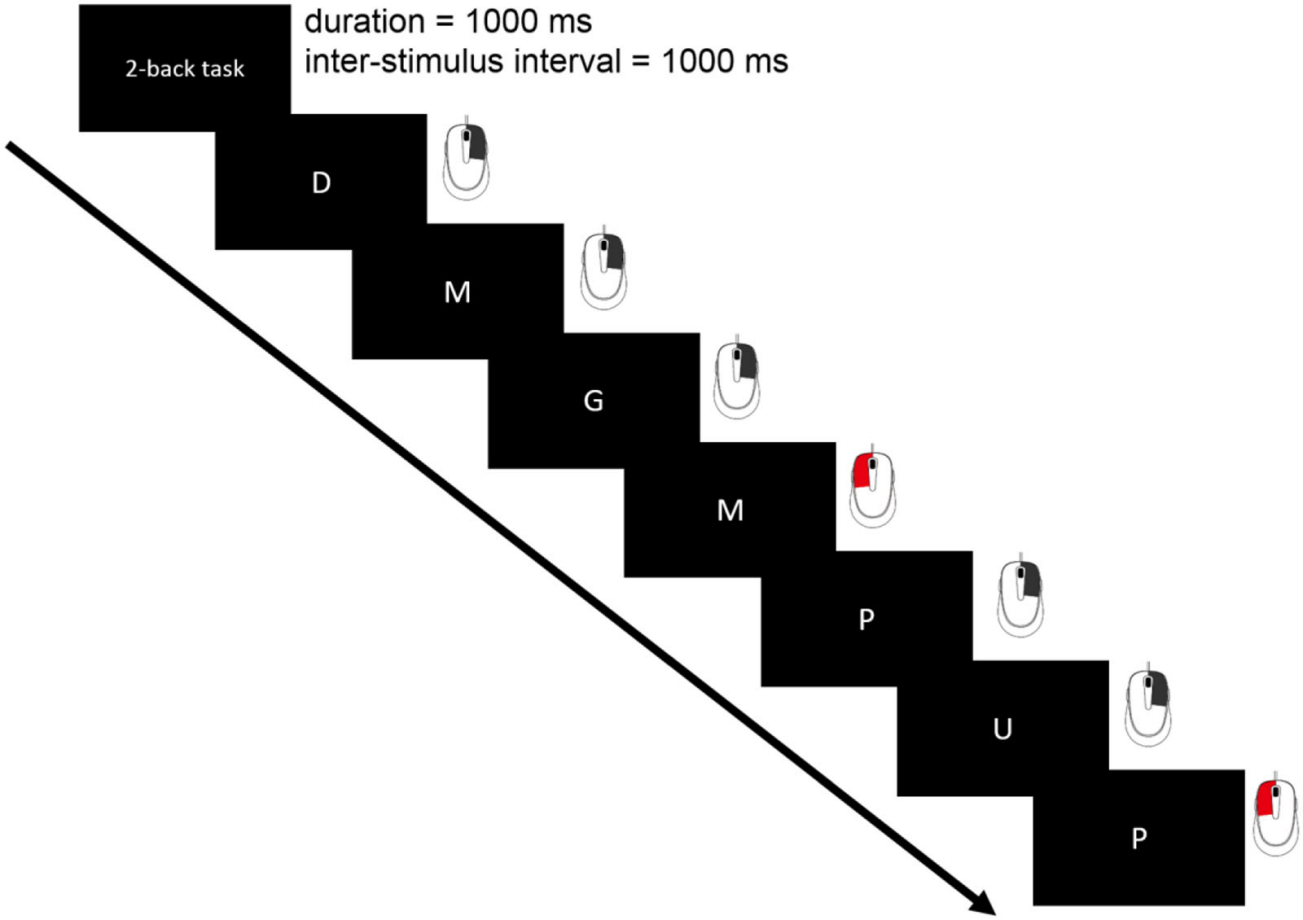
Experimental design of the letter 2‐back working memory task

Participants performed the letter 2‐back working memory task (Owen et al., 2005) as a neurobehavioral probe during EEG measurements. Using Presentation^®^, a sequence of capital letters was presented on a black background on center screen with a stimulus duration and interstimulus interval of 1000 ms. Participants were instructed to promptly click the left mouse button with their index finger if the presented letter was the same as the one presented two trials ago. Otherwise, they were instructed to promptly click the right mouse button using their middle finger (Figure 1).

### 2.5 Procedure

The participants were exposed to all the experimental (lemon, sandalwood, and kusunoki) and sham conditions at approximately 2‐min intervals. The order of inhalation conditions was counterbalanced across the participants. The distance from the display to the eyepoint was approximately 70 cm.

Participants were instructed to relax, to keep their bodies and heads still, and to fix their gaze on the screen center. After explaining the experiment protocol to the participants, the experiment was conducted as follows:

1. Rest: Participants relaxed with their eyes open for 60 s.
2. Preessential oil inhalation task: The participants performed the letter 2‐back working memory task for 2 min (60 trials).
3. Essential oil inhalation: The participants inhaled the essential oil for 2 min.
4. Postessential oil inhalation task: The participants performed the letter 2‐back working memory task for 2 min (60 trials).

There were 15 essential oil inhalation trials, each involving deep fragrance inhalation for 4 s and exhalation for 4 s through the nose; the timing of each step was displayed. In all conditions (lemon, sandalwood, kusunoki, and sham), participants rated their subjective fragrance preference immediately after inhalation using a 7‐point scale (0: undesirable to 6: preferable). Moreover, they were instructed to subjectively rate the perceived task difficulty (1: easy to 4: difficult) and concentration degree (1: unfocused to 4: focused) using a 4‐point scale after the postessential oil inhalation task.

### 2.6 EEG recording

We recorded EEG signals through BrainAmp DC (Brain Products GmbH, Munich, Germany) and Brain Vision Recorder (Brain Products GmbH, Munich, Germany) from 32 locations over the whole head (Fp1/2, F7/8, F3/4, Fz Fp1/2, F7/8, F3/4, Fz, FT9/10, FC5/6, FC1/2, T7/8, C3/4, Cz, CP5/6, CP1/2, TP9/10, P7/8, P3/4, Pz, O1/2, and Oz) based on the International 10–20 System (Klem et al., 1999) using ActiCap (Brain Products GmbH, Munich, Germany) with silver‐silver chloride active electrodes. During EEG measurement, the electrodes located at Fpz and FCz were used as the ground and system reference, respectively. The sampling frequency, time constant, and high‐cut filter were 500 Hz, 10 s, and 1000 Hz, respectively.

### 2.7 Analysis

Participant responses during the letter 2‐back working memory tasks were analyzed as behavioral data. Based on the signal detection theory, *d*' was calculated using the hit and false alarm rates and was used as a behavioral performance index (Stanislaw & Todorov, 1999).

EEG data were preprocessed offline using MATLAB 2020b (MathWorks, Inc., Natick, MA, USA) and EEGLAB (Delorme & Makeig, 2004) version 2021.0. Recorded EEG signals were band‐pass filtered at 0.15−45 Hz using a finite impulse response filter. We removed electrooculography and electromyography artifacts using the artifact subspace reconstruction (ASR) method (EEGLAB function “clean_rawdata.m”) (Mullen et al., 2015). The ASR threshold was set to 10 standard deviations from the value range recommended on the EEGLAB website (Miyakoshi, 2022, November 20), and all other parameters were turned off. We re‐referenced EEG data to a common average reference. Further, we extracted EEG data epochs for each condition as follows: rest period (1 min), preessential oil inhalation task (2 min), essential oil inhalation (2 min, 4 epochs × 30 s), and postessential oil inhalation task (2 min). We did not find any studies on the time course of the effects of essential oil inhalation on brain activity related to working memory functions. This study examined temporal changes through four epochs spanning a 2‐min period of essential oil inhalation, with 30 s (Sowndhararajan et al., 2015, 2016) as one epoch.

We estimated the intensity of cortical activity based on multichannel head‐surface EEG data using the LORETA Key software, version 20201109 (Pascual‐Marqui et al., 2011; Pascual‐Marqui et al., 1994). The head model and electrode coordinates were based on the Montreal Neurologic Institute average MRI brain (MRI152) (Fonov et al., 2011). We restricted the solution space to the cortical gray matter (6239 voxels with 5 × 5 × 5 mm spatial resolution). Finally, we calculated EEG activity in five frequency bands: delta (0.5−4 Hz), theta (4.5−7.5 Hz), alpha (8−12.5 Hz), beta (13−30 Hz), and gamma (30.5−40 Hz).

### 2.8 Statistical analysis

Statistical analyses of subjective and behavioral data were performed using SPSS, version 27 (IBM Corp., Armonk, NY, USA). The significance level was set at 5%.

To compare participants' impressions of essential oil fragrances, a Friedman test was conducted using the essential oil type and preference score as the independent and dependent variables, respectively. The Bonferroni method was used to test multiple comparisons. Similarly, we conducted Friedman tests for each subjective score with the essential oil type as the independent variable for subjective task difficulty and concentration degrees. To compare the behavioral indices in the pre‐ and postinhalation tasks in all conditions, a sign‐rank test was performed with the d' of the letter 2‐back working memory task and reaction times in the case of correct responses as dependent variables. To examine the time course of EEG activity during essential oil inhalation, current source densities during inhalation trials were calculated through each epoch (30 s) at each frequency band in all conditions and were compared to the current source density at rest (60 s). Paired *t*‐tests were used to evaluate the differences between each epoch and the rest state at each frequency. Similarly, we compared the current source density at each epoch and each frequency in the essential oil and sham conditions. Randomized statistical nonparametric mapping (SnPM) (Nichols & Holmes, 2002) was applied to correct for multiple comparisons in all current source densities and all frequencies to determine a critical probability threshold for the observed *t*‐values. A total of 5,000 permutations were performed to determine the significance in each randomization test. The critical *t*‐value (two‐tailed test) was determined as corresponding to a *p*‐value of .05.

## 3 RESULTS

**FIGURE 2:**
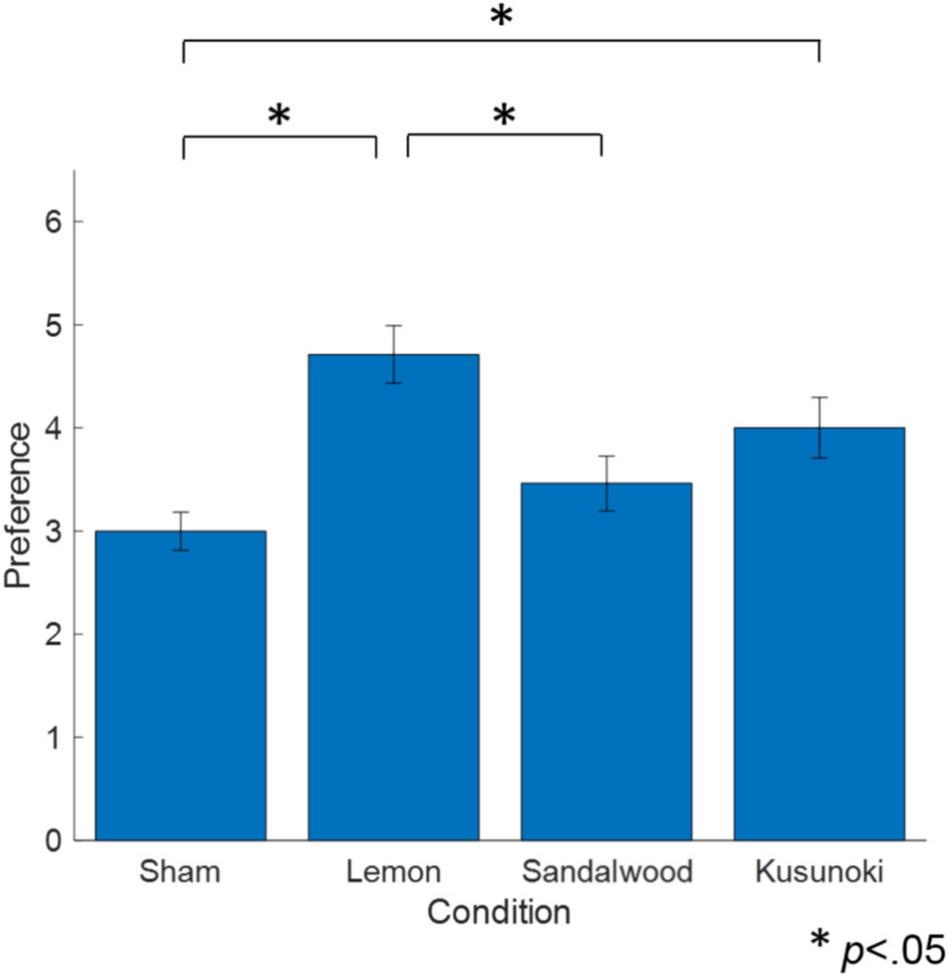
Preference score for the aroma conditions (*N* = 24, 0: undesirable to 6: preferable). The error bars represent the standard error

**FIGURE 3:**
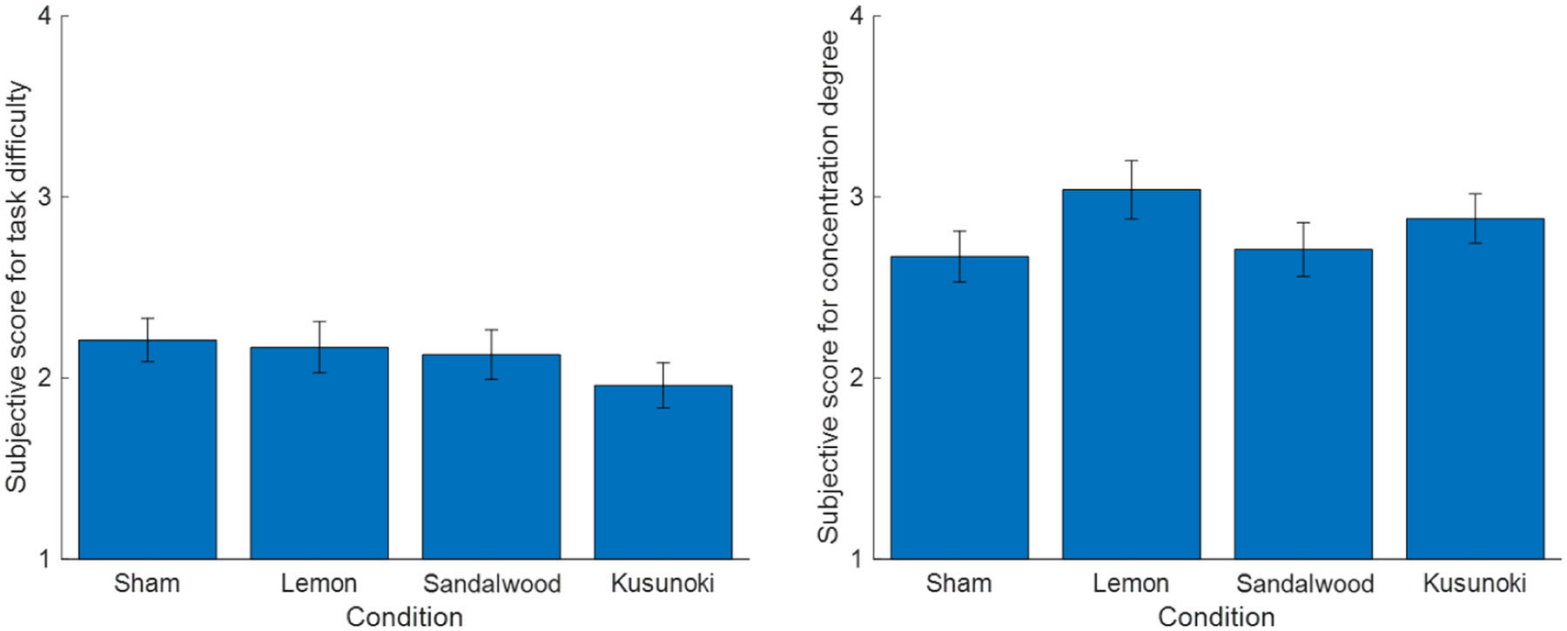
Subjective score for (left) task difficulty (*N* = 24, 1: easy to 4: difficult) and (right) concentration degree (*N* = 24, 1: unfocused to 4: focused). The error bars represent the standard error

To compare the participants' impressions of the essential oil fragrances, their subjective preferences were scored (Figure 2). A Friedman test revealed the significant main effect of each essential oil (*p* < .001). Multiple comparisons revealed a significant preference for the lemon condition compared with the sham and sandalwood ones. Kusunoki had a significantly higher preference score compared with the sham condition. The preference scores for lemon and kusunoki were high. Figure 3 shows the subjective task difficulty and concentration degree. The subjective task difficulty was the lowest for kusunoki, and the subjective concentration degree was high for lemon and kusunoki. Friedman tests of each subjective score revealed no significant differences.

**FIGURE 4:**
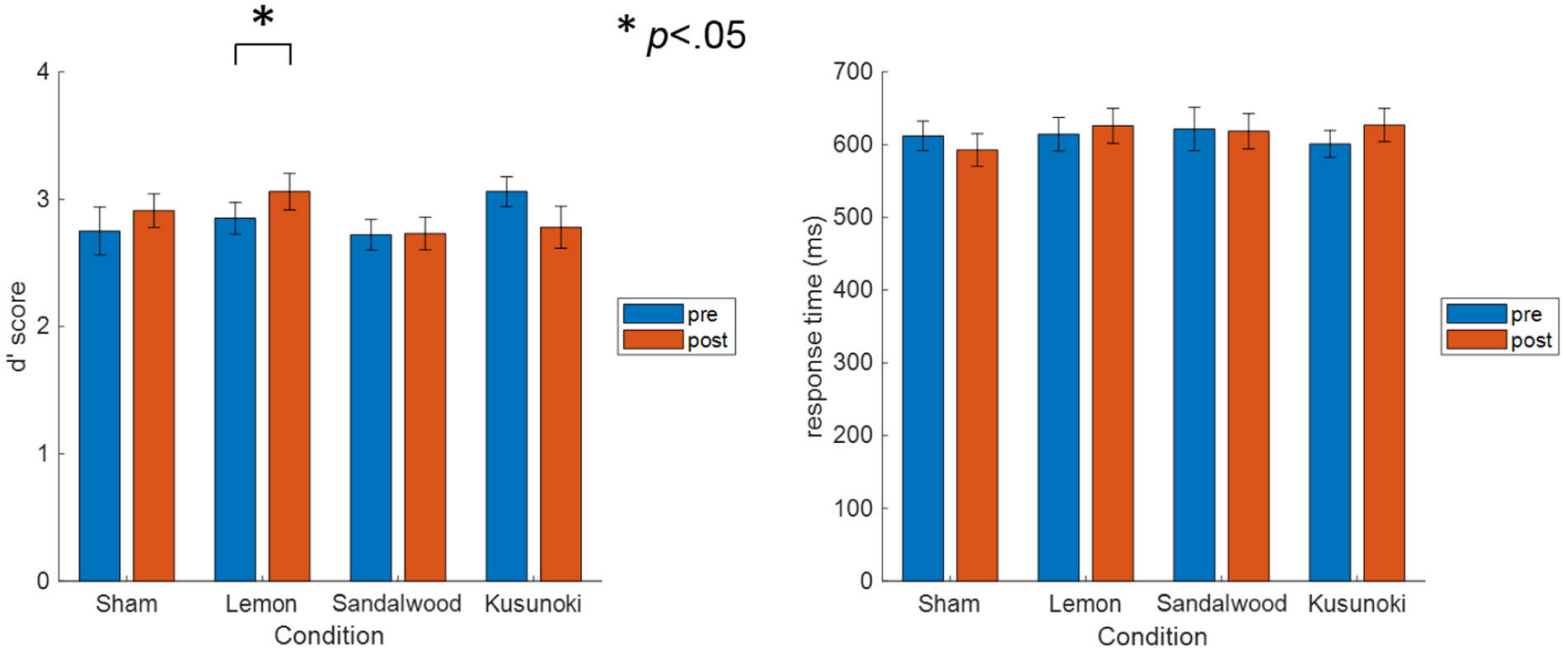
*d*’ score for the (left) 2‐back task (*N* = 24) and (right) response time in the case of correct responses (*N* = 24). The error bars represent the standard error

To compare the behavioral indices in all the conditions, we calculated the d' of the letter 2‐back working memory task and reaction times in the case of correct responses (Figure 4). A signed‐rank test revealed significantly better performance in the post‐ versus preinhalation task in the lemon condition (*p* = .035), with no significant differences in reaction times.

**FIGURE 5:**
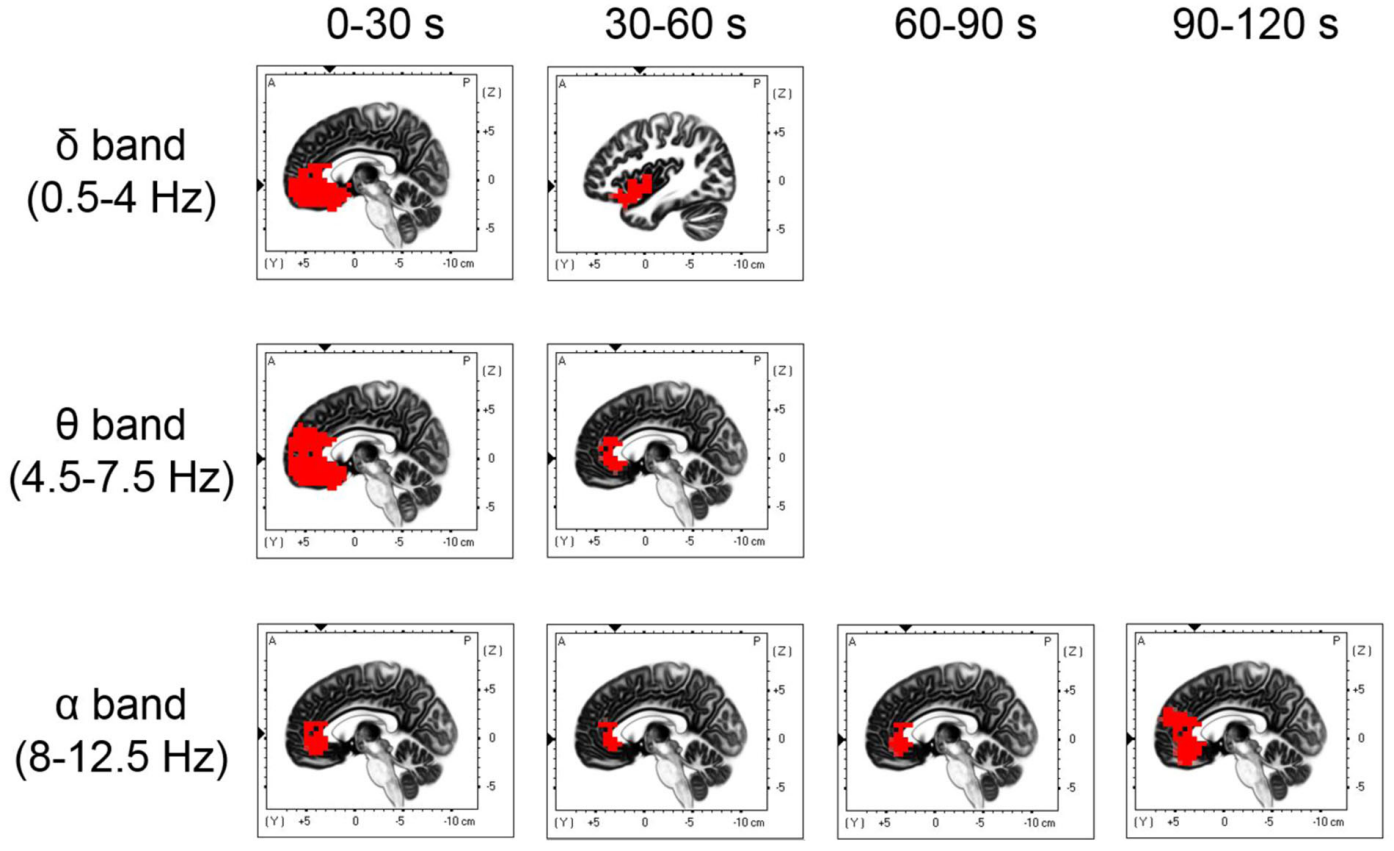
Brain regions with significantly higher activation (red areas) after lemon essential oil inhalation than in the resting state

**FIGURE 6:**
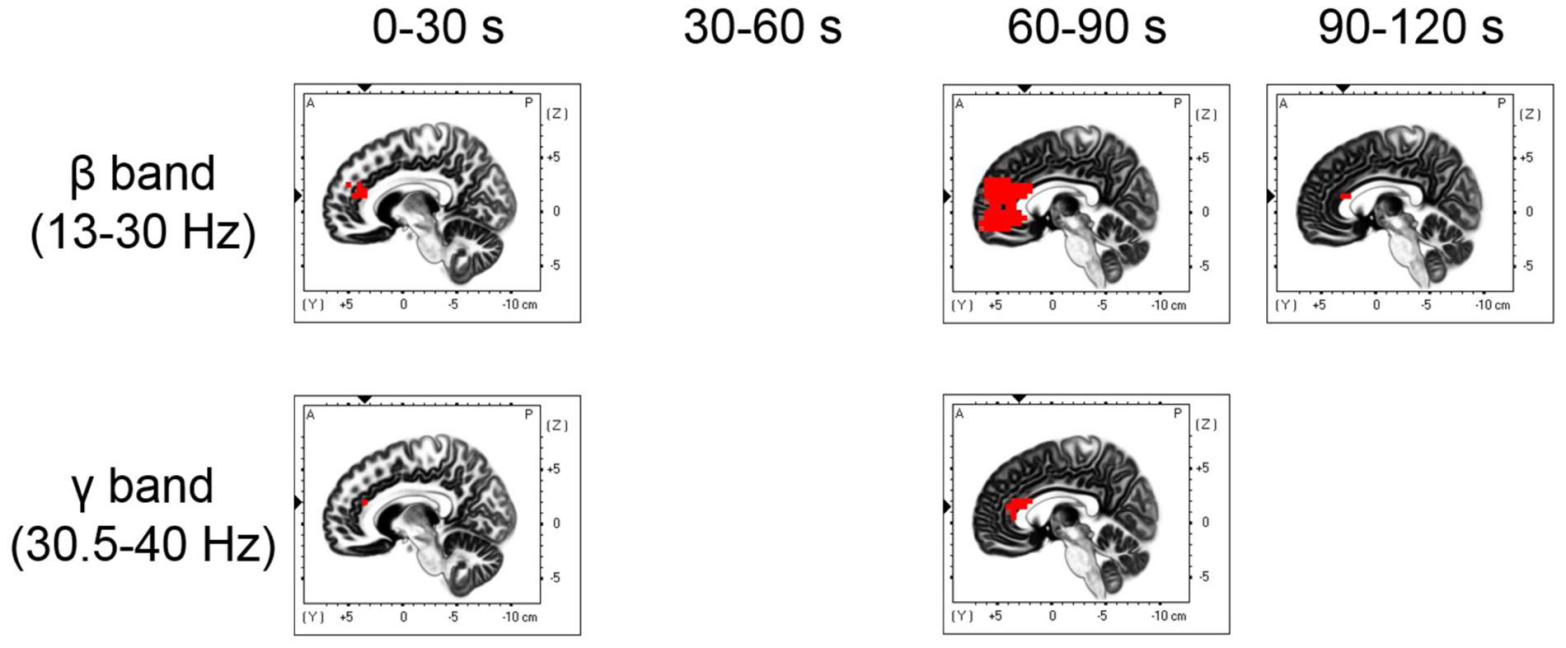
Brain regions with significantly higher activation (red areas) after sandalwood essential oil inhalation than in the resting state

**FIGURE 7:**
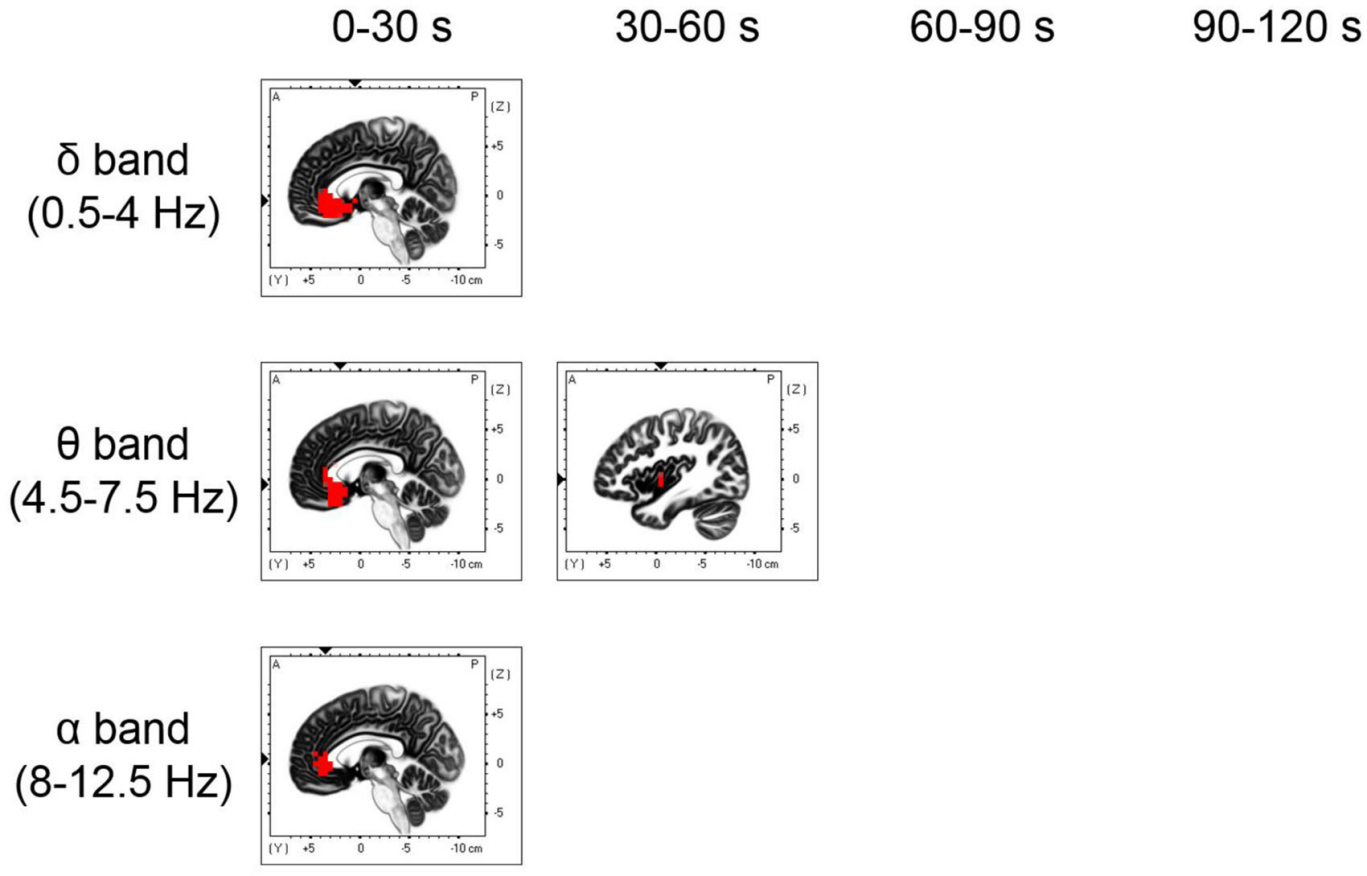
Brain regions with significantly higher activation (red areas) in the sham condition than in the resting state

To investigate the time course of EEG activity during essential oil inhalation, we calculated the current source densities during inhalation trials at 30‐s intervals for each frequency band in all conditions and compared them with the current source density during rest. Figure 5 and Table 1 show the brain regions that were significantly more active during lemon essential oil inhalation than during rest. During lemon essential oil inhalation, there was significant delta band activation in the broad prefrontal regions, including the anterior cingulate gyrus and orbitofrontal cortex, which was also seen in the superior temporal gyrus, parahippocampal gyrus, and insula between 0−30 s after inhalation onset. This activation was sustained from 30 to 60 s. Theta band activation was observed in the broad prefrontal regions, insula, and superior temporal gyrus from 0 to 30 s, and it persisted from 30 to 60 s in the prefrontal regions, such as the anterior cingulate gyrus. Alpha band activation, which lasted from 0 to 90 s after inhalation onset, was observed in the anterior cingulate gyrus and middle frontal gyrus; moreover, there was increased activation in the prefrontal cortex from 90 to 120 s. Figure 6 and Table 1 show the brain regions that were significantly more active during sandalwood essential oil inhalation compared with during rest. Significant beta band activation was found in the anterior cingulate, medial frontal gyrus, and superior frontal gyrus from 0 to 30 s after inhalation onset. Moreover, a large prefrontal region was activated from 60 to 90 s, with the anterior cingulate gyrus showing persistent activation from 90 to 120 s. Additionally, gamma band activation was seen in the anterior cingulate gyrus at 30 s after sandalwood inhalation onset and in the anterior cingulate gyrus and middle frontal gyrus at 60−90 s. Compared with the brain activity during rest, kusunoki essential oil inhalation did not significantly activate any brain region. Figure 7 and Table 1 show the brain regions that were significantly more active during inhalation of the DPG‐only samples compared with during rest. There was increased delta and theta band activation in the ventral region of the prefrontal cortex, including the anterior cingulate gyrus, supramarginal gyrus, superior temporal gyrus, and inferior parietal lobule, during the first 30 s after inhalation onset. Moreover, theta band activation was observed in the insula and superior temporal gyrus between 30 and 60 s after inhalation onset. Additionally, alpha band activation was found in the anterior cingulate gyrus and medial frontal gyrus during the first 30 s after inhalation. There was no significant activity during the pre‐ and postessential oil inhalation tasks compared with during the resting state. The EEG activity at each frequency in each essential oil condition was not significantly different from that in the sham condition at each epoch.

**TABLE 1:** Brain areas with significantly higher activation during inhalation of lemon essential oil, sandalwood essential oil, and control samples with no fragrance compared with during the resting state

		0–30 s		30–60 s		60–90 s		90–120 s	
			BA		BA		BA		BA
Lemon	δ	Anterior cingulate Medial frontal gyrus Inferior frontal gyrus Subcallosal gyrus Rectal gyrus Orbital gyrus Middle frontal gyrus Superior frontal gyrus Extra‐nuclear Superior temporal gyrus Parahippocampal gyrus Insula Uncus	10, 24, 25, 32, 10, 11, 25 11, 13, 47 11, 13, 25, 34, 47 11 11, 47 10, 11 10, 11 13, 47 38 34 47 28, 34	Insula Superior temporal gyrus Extra‐nuclear Anterior cingulate Medial frontal gyrus Subcallosal gyrus Rectal gyrus Orbital gyrus Inferior frontal gyrus Sub gyral Middle frontal gyrus Superior frontal gyrus	13, 45 13, 22, 38 13, 47 24, 25, 32 10, 11, 25 11, 13, 25, 34, 47 11 11, 47 11, 13, 47 13, 21 11, 47 11				
	θ	Anterior cingulate Medial frontal gyrus Inferior frontal gyrus Rectal gyrus Orbital gyrus Subcallosal gyrus Middle frontal gyrus Superior frontal gyrus Extra‐nuclear Insula Superior temporal gyrus	10, 24, 25, 32, 33 9, 10, 11, 25, 32 11, 13, 47 11 11, 47 11, 25, 47 10, 11 9, 10, 11 13, 47 13, 47 38	Anterior cingulate Medial frontal gyrus Inferior frontal gyrus Rectal gyrus Subcallosal gyrus	24, 32 10, 11, 25 47 11 25				
	α	Anterior cingulate Medial frontal gyrus	24, 32 10, 11	Anterior cingulate	24, 32	Anterior cingulate Medial frontal gyrus	24, 32 10, 11	Anterior cingulate Medial frontal gyrus Orbital gyrus Subcallosal gyrus Rectal gyrus Superior frontal gyrus Inferior frontal gyrus	10, 24, 32 9, 10, 11, 25 11 11 11 9, 10 11
Sandalwood	β	Anterior cingulate Medial frontal gyrus Superior frontal gyrus	32 9, 10 9			Medial frontal gyrus Superior frontal gyrus Anterior cingulate Middle frontal gyrus	9, 10, 11 9, 10, 11 10, 24, 32, 33 10	Anterior cingulate	24, 32
	γ	Anterior cingulate	32			Anterior cingulate Medial frontal gyrus	24, 32 9		
Sham	δ	Anterior cingulate Medial frontal gyrus Subcallosal gyrus Supramarginal gyrus Superior temporal gyrus Inferior parietal lobule Inferior frontal gyrus Rectal gyrus	24, 25, 32 10, 11, 25 25, 47 40 22 40 47 11						
	θ	Anterior cingulate Medial frontal gyrus Subcallosal gyrus Rectal gyrus Inferior frontal gyrus	24, 25, 32 11, 25 11, 13, 25 11 47	Insula Superior temporal gyrus	13 22				
	α	Anterior cingulate Medial frontal gyrus	24, 32 10, 32						

BA: Brodmann area, δ: 0.5−4 Hz, θ: 4.5−7.5 Hz, α: 8−2.5 Hz, β: 13−30 Hz, γ: 30.5−40 Hz. The brain regions are shown in descending order of activity in each period and frequency.

## 4 DISCUSSION

We investigated the effects of inhaling lemon, sandalwood, and kusunoki essential oils on human brain activity and memory function, including those in deep brain regions, using multichannel EEG and brain source activity estimation. Participants were required to complete the letter 2‐back working memory task before and after essential oil inhalation; the results were used to examine the time course of brain activation as well as the changes in subjective and behavioral measures after essential oil inhalation.

Lemon essential oil was preferred over DPG‐only samples and sandalwood oil. Additionally, kusunoki essential oil was preferred over DPG‐only samples. Inhalation of lemon essential oil enhances positive mood (Kiecolt‐Glaser et al., 2008); furthermore, a subjective preference for the fragrance of lemon essential oil may induce a positive mood. Similar to in the case of lemon oil, the subjective preference for the fragrance of kusunoki essential oil may induce a positive mood. Future studies should elucidate the subjective effects of kusunoki essential oil inhalation.

We examined the pre‐ and postinhalation working memory task performance using d' calculated from the hit and false alarm rates of the responses and reaction time in the case of correct responses. There were no significant among‐condition differences in reaction time; however, d' was significantly higher after lemon essential oil inhalation compared with before, which indicated improved working memory performance.

Lemon oil can facilitate word recall in humans (Berg, 1987). A rat study reported that lemon essential oil inhalation reduced scopolamine‐induced memory impairment (Zhou et al., 2009). Additionally, the use of lemon fragrance in an environmental fragrance system functioning through a building's central air conditioning system significantly reduced key entry errors among video display operators (Manley, 1993). This suggests that lemon essential oil inhalation may enhance working memory function and concentration.

We observed delta and theta band activation in the prefrontal cortex, including the anterior cingulate gyrus and orbitofrontal gyrus, superior temporal gyrus, parahippocampal gyrus, and insula from 0 to 60 s after inhalation onset of lemon essential oil. Moreover, alpha band activation was observed in the prefrontal cortex, including the anterior cingulate gyrus, immediately after inhalation, with a broad prefrontal region exhibiting activation from 90 to 120 s. Furthermore, beta band activation was seen in the prefrontal regions, including the anterior cingulate gyrus, from 0 to 120 s after inhalation onset of sandalwood essential oil. Finally, gamma band activation was observed in the anterior cingulate gyrus immediately after inhalation and in both the anterior cingulate gyrus and medial frontal gyrus from 60−90 s.

The orbitofrontal cortex receives all sensory information (visual, auditory, tactile, olfactory, and gustatory) (Rolls, 2004), reproduces it, predicts the outcome of the ensuing action, and formulates a behavioral strategy (Rudebeck & Murray, 2014). Specifically, the orbitofrontal cortex processes olfactory information for odor identification and reward value. It has dense fibrous connections with the limbic system, including the ventral region of the anterior cingulate gyrus and anterior insula, which were activated in our study. Additionally, emotional processing occurs within the network of the orbitofrontal cortex, anterior cingulate gyrus, and insula (Bush et al., 2000). Inhaling lemon essential oil may induce a subjective preference response through emotional processing in the aforementioned network.

Oscillatory activity in the frontal lobes within the low‐frequency range (delta to theta) is essential for top‐down control of endogenous attentional selection during working memory tasks (Daitch et al., 2013; Jensen & Tesche, 2002; Johnson et al., 2017; Onton et al., 2005; Sauseng et al., 2010, 2005). These rhythms potentially represent the temporal basis of information processing required for memory encoding and retrieval processes (Hsieh & Ranganath, 2014; Jensen & Tesche, 2002; Mormann et al., 2008). Specifically, the frontal midline theta band is actively involved in the encoding, maintenance, and recall of working and episodic memory (Hsieh & Ranganath, 2014; Jensen & Tesche, 2002). There are similar frontal alpha and theta oscillations involved in working memory, which have similar functions in controlling working memory (Meltzer et al., 2008; Sauseng et al., 2010). Further, frontal beta (Tallon‐Baudry et al., 1999) and gamma (Roberts et al., 2013; Roux & Uhlhaas, 2014) band activity is involved in memory maintenance. Our results suggest that delta band activation in the prefrontal cortex and parahippocampal gyrus, as well as theta and alpha band activation in the prefrontal cortex, during lemon essential oil inhalation after a working memory task contributes to memory encoding and retrieval, as well as to the control of attentional selection. This eventually improves performance in the working memory task after inhalation. Moreover, the delta and theta bands were activated immediately after lemon essential oil inhalation. The alpha band was continuously activated after inhalation, and there was a persistent increase in the activity. It is unclear which of the brain activities observed during lemon essential oil inhalation in this study contributed the most to subsequent working memory performance, but future experiments performed with different inhalation durations should clarify this. Beta‐ and gamma band activation in the prefrontal cortex immediately after sandalwood essential oil inhalation may facilitate memory maintenance. Specifically, there was a sustained significant increase in beta band activity. However, sandalwood oil did not significantly improve task performance after inhalation. Therefore, there is a need for future studies to elucidate the relationship between brain activation induced by inhalation and behavioral performance.

Brain activations were observed immediately after both lemon and sandalwood inhalation. The activations associated with lemon essential oil inhalation were high immediately after inhalation, and alpha‐band activity was maintained at a high level. Contrarily, brain activations increased with a time delay after sandalwood essential oil inhalation. These findings suggest that the appropriate inhalation duration that contributes to brain activation differs among essential oils.

The mechanism through which essential oil inhalation after a working memory task activates brain regions involved in working memory remains unclear. Essential oils are absorbed into the human body through three main routes: oral, dermal, and nasal. Upon inhalation, they are absorbed and broken down in the lungs, from where they enter the bloodstream and circulate throughout the body. Alternatively, odor molecules bind to olfactory receptors, sending signals to the brain. When examining the effects of essential oil fragrances on various human functions, it is important to determine whether odor molecules entering the bloodstream reach the cerebrum. The blood‐brain barrier (BBB) restricts the transfer of substances from the blood to the brain (Kandel et al., 2012). Essential oil odor molecules, which are fat soluble, can cross the BBB and reach the cerebrum (Hongratanaworakit, 2011), suggesting that essential oil fragrances affect cognitive functions neurologically. During the task, there is increased regional blood flow in active areas of the cerebral cortex. In case this blood flow is maintained after the task, odor molecules entering the bloodstream after essential oil inhalation may be transported to these active areas where they enhance neural activity. Accordingly, odor molecules are actively transported to task‐related brain regions, selectively activating these regions. Neuropharmacological studies are needed to clarify the temporal mechanism of action of these molecules.

Based our findings, it is expected that essential oil inhalation can be used as an intervention in patients with cognitive dysfunction, especially memory dysfunction, on considering the type of essential oil and duration of essential oil inhalation. In the future, it is necessary to investigate the effects of essential oil inhalation on various cognitive functions (e.g., attention, memory, language) and brain activities.

This study has several limitations. First, kusunoki essential oil inhalation was associated with higher subjective preference for fragrance, lower working memory task subjective difficulty, and higher postinhalation concentration degree. However, no significant brain activation was seen during inhalation. This could be attributed to the kusunoki essential oil concentration and inhalation time duration. Future studies should investigate the cognitive effects by altering the essential oil inhalation parameters and employing other cognitive tasks, including attention‐related tasks. Second, no significant brain activation was observed during the working memory task compared with during rest. This could be attributed to the low difficulty of the letter 2‐back working memory task given the relatively low subjective difficulty reported by the participants. Moreover, it could also be attributed to the activation of prefrontal regions, including the medial prefrontal cortex, during both the resting state and working memory task. Brain regions in the default mode network, including the medial prefrontal area, are activated during the resting state (Chen et al., 2008). Brain activity during the task pre‐ and postessential oil inhalation must be investigated by changing the difficulty of the working memory task. Third, we observed prefrontal cortex activation during the inhalation of the DPG‐only sample; however, the activation area was smaller than that during lemon essential oil inhalation. Brain activity in each essential oil condition was not significantly different from that in the sham condition. These findings are suggestive of activation (Sarnthein et al., 1997) immediately after working memory task performance or of the effects of psychological factors, including expectation (i.e., placebo effect) (Babulka et al., 2017). Further research with experiments involving other cognitive function tasks is required. Fourth, this experiment included participants without self‐reported abnormalities in the olfactory senses, and no olfactory threshold test was conducted. In order to minimize the risk of bias, future studies should validate the results using an olfactory threshold test to ensure homogeneity in olfactory performance across participants. Finally, we only included men in this study. There are reported sex‐based differences in the effects of essential oils on EEG activity (Chandharakool et al., 2020). Future studies should also investigate the cognitive effects of essential oil inhalation in women.

## 5 CONCLUSION

We investigated the effects of inhaling lemon, sandalwood, and kusunoki essential oils on human brain activity and memory function, including those in deep brain regions, using multichannel EEG and brain source activity estimation. This is the first study to report the activation of brain regions involved in emotional and memory processing through essential oil inhalation and time course of activation. Our findings will contribute to further understanding the effects of essential oil products on cognitive functions and brain activity and can aid in developing scent products for patients with impaired memory and emotional functions and healthy individuals seeking to adaptively maintain and promote memory and emotional functions. Future studies should clarify the effects of inhaling other essential oils on other cognitive functions and underlying brain mechanisms.

## CONFLICT OF INTEREST

This study was funded by Nippon Kodo Co., Ltd. TH and TS are employees of Nippon Kodo Co., Ltd.

### ETHICS STATEMENT

This study was approved by the Ethics Committee of the Graduate School of Engineering, The University of Tokyo (approval number: KE20‐85) and was conducted in accordance with the Declaration of Helsinki.

### PATIENT CONSENT STATEMENT

All participants provided written informed consent for participation.

### PEER REVIEW

The peer review history for this article is available at https://publons.com/publon/10.1002/brb3.2889

## Data Availability

The data that support the findings of this study are available from the corresponding author upon reasonable request.
